# Cost-of-illness comparison between clinical judgment and molecular point-of-care testing for influenza-like illness patients in Germany

**DOI:** 10.1038/s41533-022-00325-4

**Published:** 2023-01-17

**Authors:** M. Brachmann, P. Serwa, D. Sauerland

**Affiliations:** 1 Witten/Herdecke University, Witten, Germany; 2 bcmed GmbH, Ulm, Germany

**Keywords:** Health care economics, Public health, Respiratory tract diseases

## Abstract

A high economic burden stems from seasonal influenza as a well-known but serious public health problem. Rapid diagnostic tests have not yet been integrated into routine use in German primary care, even though they are likely to reduce overall costs in cases of suspected infection. This study aims to demonstrate that the use of point-of-care testing (POCT) produces lower costs of illness compared to the costs incurred by relying on clinical judgment alone. With the help of a decision tree model, two different diagnostic approaches for influenza-like illness (ILI) in primary care were compared: (1) clinical judgment with no technical support and (2) POCT. The costs of illness, as well as their differences, vary widely among the three age groups considered (elderly people, adults, and children). For the pathway of using clinical judgment alone, the costs of illness sum up to 155.99 € for elderly people compared to 76.31 € for adults and 74.15 € for children. With POCT, the costs of illness for the elderly amount to 115,09 €, which is 26% lower than the costs without diagnostic support. The costs for adults and children are 74.42 € and 75.66 €, respectively, which means 2.5% lower costs of illness for adults and 2% higher costs for children. The results demonstrate that the use of POCT to support detecting influenza in ILI patients may reduce the overall cost of illness. The provided data can help governments make informed decisions about potential cost savings by integrating POCT into the reimbursement scheme.

## Introduction

Influenza is a highly transmissible, acute infection of the respiratory system, causing seasonal epidemics, especially during winter months. It can lead to numerous complications, such as chronic cardiac, pulmonary, or metabolic diseases^1^. Influenza is a significant contributor to global mortality and morbidity, causing approximately 500,000 deaths per year worldwide^2^. In the 2017/2018 influenza season in Germany, approximately 9 million patients were registered as seeking medical care in primary healthcare settings due to influenza-related diseases. A total of 60,000 patients were registered with influenza-related hospitalizations. The public health department reported influenza-related deaths in 1129 cases^3^. These data indicate a serious public health problem causing a high economic burden for society.

According to the WHO Manual (2016), the economic burden of a disease from a societal perspective is the sum of direct and indirect costs. Direct costs are associated with the use of medical resources (e.g., ambulatory care, hospitalization, and pharmaceuticals) usually covered by the health system payers and with individual expenditures by the patient or patient’s families (e.g., transportation to the hospital, additional food costs, and additional expenses for accommodation). Indirect costs are defined mainly as the value of lost productivity because of absenteeism from work or school that applies to patients and their caregivers during disease-related treatment and hospitalization^4^. Influenza-like illnesses (ILIs) contribute to loss of productivity as an indirect cost that makes up 80–90% of the total costs of illness^5^. The direct cost of illness, together with the productivity loss in Germany, sum up to 2.2 billion Euro per year in an average season and can even be higher in peak seasons^6^. The corresponding cost per confirmed influenza case was 514 € from a societal perspective and 59 € from a health systems payer perspective in 2012^7^.

It has already been shown that the routine use of rapid influenza tests and early diagnosis is likely to reduce overall costs in cases of suspected influenza in German hospitals^8^. The detection of influenza during a patient’s first visit to primary care allows immediate clinical decisions, including targeted antiviral treatment and infection control measures^9^. Early antiviral therapy reduces the risk of hospitalization, complications, or even death^10^. Early detection also reduces inappropriate use of antibiotics and decreases additional GP visits as well as additional diagnostics (e.g., bloodwork or chest X-rays)^11,12^. Timely and accurate diagnosis is also a key aspect in the prevention of virus spread^13^. However, distinguishing influenza from other respiratory viruses causing similar symptoms is a difficult task for physicians^14^. Up-to-date point-of-care testing (POCT) can help to overcome this problem. Therefore, countries such as Japan have established and integrated POCT as a standard procedure in their diagnostic pathway for influenza in primary care^15^.

In the past, rapid diagnostic tests for influenza were not very reliable because of their low sensitivity of antigen detection^16^. In recent years, many more dependable new rapid nucleic acid amplification tests (NAATs) have been developed. They are self-contained, easy to use, and can detect viral RNA in 15 min. In Germany, POCT for influenza is not yet part of the reimbursement scheme of the statutory health insurance and hence is not part of standard diagnostics.

The objective of this study is to analyze differences in the cost of illness when using a diagnostic pathway supported by molecular POCT in comparison to clinical judgment in primary care in Germany. Estimates on this issue have not been published thus far. Providing data on potential cost savings can help governments make an informed decision about the potential benefits of integrating POCT into the reimbursement scheme of SHI funds.

## Methods

**Fig. 1:**
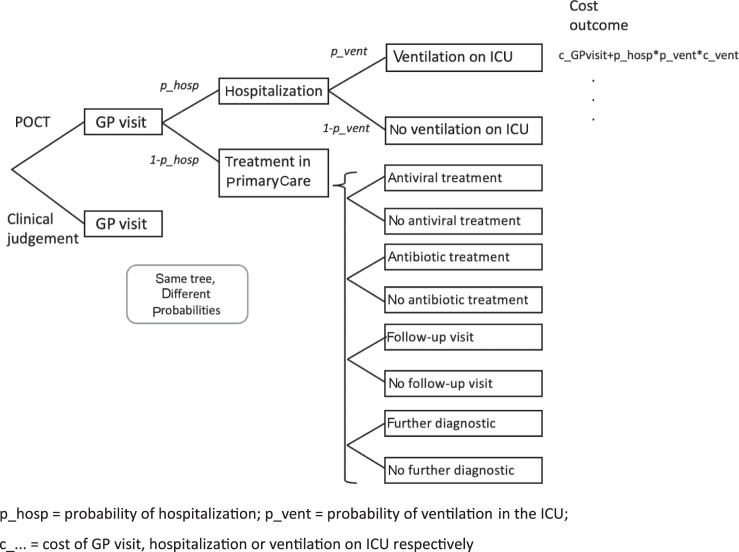
Simplified cost-of-illness calculation model. The figure shows the decision tree and simplified cost calculation model that was developed to simulate ILI patients in primary care with the two different diagnostic approaches.

As a direct cost-of-illness calculation, this study compared NAAT POCT and clinical judgment to diagnose influenza from a healthcare system perspective. The definition of direct costs followed the WHO Manual for estimating the economic burden of seasonal influenza^4^. A decision tree model (Fig. 1) was developed to simulate ILI patients in primary care with different diagnostic approaches. Patients directly presenting in an emergency department or an urgent care center were excluded.

There are two paths in the model. One depicts the patient’s path when seeing a physician using rapid testing as diagnostic support and represents the status quo of ILI treatment in Germany. The other path follows patients with only clinical judgment as a diagnostic approach. Both paths have corresponding consequential costs depending on the different probabilities of resource utilization and the prescribed therapy. The sum of the costs of each path represents the direct costs of illness for the diagnostic approach. To display the change in the probability of resource utilization, such as hospitalization or follow-up visits, odds ratios were used. A systematic literature search in MEDLINE was conducted to retrieve literature in which healthcare resource utilization with and without POC diagnostics was measured. The keywords used were “influenza”, “cost of illness”, “hospitalization”, “point-of-care testing”, and “early treatment”. The studies were selected based on the following criteria: (1) publications were written in English and German, (2) rapid tests were performed with NAAT, and (3) the study was performed in an OECD country. Furthermore, only studies published between 2010 and 2021 were selected. Since POC influenza testing is not yet common in primary care, the search was expanded to secondary care studies as well, in cases where there were no original data available from primary care. Whenever the studies used age as a discriminating factor was taken over into this study as well. All articles found were screened for relevance and applicability to the present analysis. Whenever variables were found in several publications, the weighted average was used in the calculation for the cost-of-illness calculation.

### Model inputs

Clinical parameters are shown in Table 1. This was a retrospective study that was conducted by analyzing database medical records of patients diagnosed by German office-based physicians in two influenza seasons from May 2010 to April 2012. The probability of additional GP visits was estimated to be 20% for adults and 50% for children, the probability of antiviral treatment was 5.7% for adults and 6.6% for children, and the probability of antibiotic treatment was 33.1% for adults and 16.8% for children^7^. A paid transport to GP probability of 6.5% was calculated based on the number of total paid transports of nonemergency ambulance rides as well as paid taxi transports (46 million)^17^ and the number of 709 million total visits in primary care^18^. Influenza-related hospital admissions based on data from German primary care practices from the 2017/2018 season were reported for adults (0.31%), children (0.3%), and elderly people (1.5%)^3^. The additional X-ray probability was reported to be 33 and 52% for other diagnostics in a randomized trial conducted in a pediatric emergency department and an acute care clinic^19^.

**Table 1:** Clinical parameters.

	Adults		Children		Elderly people		References
Pathway	Clinical judgment (%)	POCT (%)	Clinical judgment (%)	POCT (%)	Clinical judgment (%)	POCT (%)	
*Clinical parameters*							
P additional GP visit	20	8	50	20	20	8	^11^
P paid transport to GP	6.5	6.5	6.5	6.5	6.5	6.5	^17,18^
P paid transport to the hospital	58	58	58	58	58	58	^29^
P antiviral treatment	5.70	11.97	6.60	13.86	5.70	11.97	^11^
P antibiotic treatment	33.10	19.86	16.80	10.08	33.10	19.86	^11^
P hospitalization *(p_hosp)*	0.31	0.16	0.30	0.15	1.50	0.75	^8^
P ventilation in the ICU when hospitalized *(p_vent)*	7	7	7	7	7	7	^24^
P X-ray	33	20	0	0	33	23	^19^
P other diagnostics	52	39	52	39	52	39	^19^

The cost parameters are shown in Table 2. The economic model calculation was conducted from the perspective of healthcare payers in Germany; therefore, pricing in local currency was used. Costs for different medical services found in reports or scientific literature, if obtained from different years than 2020, were adjusted to the base year 2020 for the inflation rate in Germany based upon the consumer price index. Costs for the initial GP visit were taken from the reimbursement catalog of the National Association of Statutory Health Insurance Physicians^20,21^ and depended on the patient age group: 13.20 € for adults, 20.89 € for children, and 19.92 € for elderly people. Since that payment is a quarterly lump sum, the costs for follow-up visits of patients can only be taken into account if that visit happens in the next quarter. Given a uniform distribution of patient visits in a quarter and a maximum interval of 7 days for the next visit, only 4% of the follow-up visits are reimbursed and hence included in the follow-up costs. Testing costs for NAAT testing of influenza A and B are also provided in the catalog and listed at 16.50 €. As already mentioned, GPs currently are not reimbursed for POCT with ILI patients. Based on the database of the National Association of Statutory Health Insurance, the cost of transportation was taken from 2018 and inflation-adjusted estimated to be 44.27 € in 2020^17,22^. The daily cost of antivirals of 7.66 € and 1.44 € for antibiotics for 2020 was used based on the pricing from ifap Service - Institute for Physicians and Pharmacists GmbH^23^. Costs per hospital stay were taken from an influenza-specific German hospital cost calculation based on representative data of publicly insured patients, including ICD-10 diagnosis and hospitalization cost data, in the period of 2013–2019. The data distinguish patients with and without ventilation in the ICU during their hospital stay. The costs add up to 36,000.00 € for patients ventilated in the ICU and 3,400.00 € for patients treated in normal wards^24^. Also included were reimbursable transportation costs of 452.77 € for patients coming to the hospital in an ambulance^17,22^. Chest X-ray costs of 16.24 € were complemented by an age-specific lump sum for each patient, both of which are taken from the German reimbursement catalog^20^. Costs for other diagnostics of 56.85 € were taken from a Spanish POCT study^25^.

**Table 2:** Cost inputs.

Cost inputs (EUR)	Adults	Children	Elderly people	References
Costs per GP visit	13.20	20.89	19.92	^20^
Costs per GP follow-up visit	0.5	0.79	0.76	Calculation based on KBV^20^ and probability of 3,8% to see a physician in the following quarter
Testing costs	16.5	16.5	16.5	^20^
Costs of transport to the GP	44.27	44.27	44.27	^17,22^
Costs of antivirals/day	7.66	7.66	7.66	^23^
Costs of antibiotics/day	1.44	1.44	1.44	^23^
Costs per hospital stay (patients ventilated in the ICU) *(c_vent)*	36,000	36,000	36,000	^24^
Costs per hospital stay (patients nonventilated in the ICU) *(c_hosp)*	3,400	3,400	3,400	^24^
Reimbursable costs of transport to the hospital	452.77	452.77	452.77	Own calculation based on the number of missions^17^ and total mission costs^22^
Costs per X-ray	24.36	23.03	24.36	^20^
Costs for other diagnostics	56.85	56.85	56.85	^25^

The distinction in costs of illness mainly stems from different probabilities in the follow-up treatment of ILI patients. As described above, those probabilities were taken from the literature in which the effect of POCT on follow-up resource utilization was examined. With influenza POCT in ILI patients, the probability of receiving antiviral medication is more than twice as high as that with the use of clinical judgment. This probability is, on the other hand, lower (0.6) for an antibiotic prescription. Since early testing provides the first diagnostic guidance, the odds ratios for additional diagnostics as well as follow-up visits are also lower than 1. The most cost-intensive resource in health care, hospitalization, is also strongly influenced by the adoption of rapid testing. The odds ratio of the hospitalization rate was 0.5 when comparing POCT with clinical judgment. When physicians rely on clinical judgment alone to diagnose a patient, it is twice as likely that the patient will be hospitalized. The described ratios are summarized in Table 3. They are used for calculating the changes in the probability of resource utilization for the POCT approach compared to clinical judgment and are reflected in the clinical parameters shown in Table 1.

**Table 3:** Differences in probabilities for resource utilization comparing clinical judgment and POCT.

	Odds ratios: clinical judgment and POCT	Sources
Antiviral prescription	2.1	^30^
Antibiotic prescription	0.6	^5,30^
X-rays	0.7	^19^
Other diagnostic tests	0.8	^19^
Additional GP visit	0.4	^5^
Hospitalization	0.5	^31^

## Results

### Overview and composition of costs

The cost of illness for ILI patients presenting in primary care in Germany without a POCT-assisted diagnosis is 87.87 € (see Table 4). The three largest cost factors are additional diagnostics, the first GP visit, and the costs of hospitalization. They add up to 86% of the total costs of illness. This lies within the scope of a prior study calculating the economic burden of influenza in which direct costs added up to an inflation-adjusted 63.03 €^26^. That study is from 1999 and does not include transport costs or antiviral medication. Using molecular POCT to detect influenza in ILI patients, the direct costs add up to 80.83 € (see Table 4). The first two largest cost factors are the same as those in the clinical judgment approach, but the third one, namely testing costs, is unique to the POCT pathway. Together, the three sum up to 67% of the total costs. Comparing both pathways, 8% or 7.05 € lower costs for patients diagnosed with POCT can be found.

**Table 4:** Calculation (general).

Cost item	POCT	Clinical judgment	Delta	Delta p.p.
Antivirals	43,066,197.34 €	20,458,870.96 €	−22,607,326.38 €	−2.48 €
Antibiotics	23,230,593.64 €	38,620,117.79 €	15,389,524.15 €	1.69 €
Hospitalizations	137,952,124.19 €	272,360,682.54 €	134,408,558.35 €	14.76 €
GP: 1st visit	144,509,610.37 €	144,509,610.37 €	− €	− €
GP: follow-up visits	726,132.24 €	1,811,177.87 €	1,085,045.63 €	0.12 €
X-ray	38,190,847.07 €	54,646,839.36 €	16,455,992.29 €	1.81 €
Additional diagnostics	198,189,706.91 €	267,932,342.54 €	69,742,635.63 €	7.66 €
Testing costs	150,278,700.00 €	- €	−150,278,700.00 €	−16.50 €
Sum	736,143,911.76 €	800,339,641.43 €	64,195,729.67 €	7.05 €
Cost of illness p.p.	80.83 €	87.87 €		

### Detailed costs of illness

When comparing the costs of illness in detail, a rise in antiviral medication costs of 2.48 € became apparent when using molecular POCT. This result is not surprising since the precise prescription of antivirals is one of the main advantages of using POCT for detecting influenza infection. The opposite effect can be observed with an antibiotic prescription: The costs drop by 1.69 € when POCT is implemented. In relative savings in the number of prescriptions, the additional antivirals are nearly offset by the reduction in antibiotics. In absolute monetary terms, however, antiviral medication is more costly than widely available antibiotics. In addition, targeted use of medication and prevention of unnecessary antibiotic prescriptions may have even a greater benefits than only decreasing health care costs directly, as it helps in reducing the problem of antimicrobial resistance. Other aspects with lower costs on the POCT diagnostic pathway are hospitalizations as well as a reduced volume of additional diagnostics.

### Differences between the age groups

The costs of illness differ widely among the age groups (see Tables 5–7 for age-specific results). Using clinical judgment alone, the costs of illness sum up to 155.99 € for elderly people (aged 60+) compared to 76.31 € for adults and 74.15 € for children. With POCT, the costs of illness for elderly people amount to 115,09 €, which is 26% lower than the costs without diagnostic support, as displayed in Table 7. The costs for adults and children, as depicted in Tables 5 and 6, are 74.42 € and 75.66 €, respectively, which means 2.5% lower costs of illness for adults and 2% higher costs for children. For the elderly population, the largest cost factor is hospitalization, and the main cost driver for adults and children is GP visits. Since this visit cannot be reduced with POC diagnostics, the hospitalization rate is lowered for ILI patients when not only using clinical judgment.

**Table 5:** Calculation (age-group specific) children.

Cost item	POCT	Clinical judgment	Delta	Delta p.p.
Antivirals	10,372,916.91 €	4,932,063.89 €	−5,440,853.02 €	−2.78 €
Antibiotics	2,836,363.70 €	4,720,171.28 €	1,883,807.57 €	0.96 €
Hospitalizations	18,008,617.34 €	36,017,234.67 €	18,008,617.34 €	9.20 €
GP: 1st visit	40,881,732.79 €	40,881,732.79 €	− €	− €
GP: follow-up visits	351,646.80 €	877,796.35 €	526,149.55 €	0.27 €
X-ray	− €	− €	- €	− €
Additional diagnostics	43,324,541.06 €	57,679,275.50 €	14,354,734.44 €	7.34 €
Testing costs	32,290,500.00 €	- €	−32,290,500.00 €	−16.50 €
Sum	148,066,318.60 €	145,108,274.47 €	−2,958,044.13 €	−1.51 €
Cost of illness p.p.	75.66 €	74.15 €

**Table 6:** Calculation (age-group specific) adults.

Cost item	POCT	Clinical judgment	Delta	Delta p.p.
Antivirals	26,438,676.99 €	12,570,931.15 €	−13,867,745.84 €	−2.40 €
Antibiotics	16,492,577.40 €	27,446,331.47 €	10,953,754.08 €	1.90 €
Hospitalizations	56,697,053.56 €	109,850,541.27 €	53,153,487.71 €	9.20 €
GP: 1st visit	76,245,842.79 €	76,245,842.79 €	− €	− €
GP: follow-up visits	280,303.23 €	699,705.25 €	419,402.02 €	0.07 €
X-ray	30,547,000.25 €	43,762,456.90 €	13,215,456.64 €	2.29 €
Additional diagnostics	127,862,111.07 €	170,226,680.72 €	42,364,569.65 €	7.33 €
Testing costs	95,307,300.00 €	− €	−95,307,300.00 €	−16.50 €
Sum	429,870,865.28 €	440,802,489.55 €	10,931,624.27 €	1.89 €
Cost of illness p.p.	74.42 €	76.31 €

**Table 7:** Calculation (age-group specific) elderly people.

Cost item	POCT	Clinical judgment	Delta	Delta p.p.
Antivirals	6,254,603.44 €	2,955,875.92 €	−3,298,727.52 €	−2.40 €
Antibiotics	3,901,652.54 €	6,453,615.04 €	2,551,962.49 €	1.86 €
Hospitalizations	63,246,453.30 €	126,492,906.60 €	63,246,453.30 €	46.01 €
GP: 1st visit	27,382,034.79 €	27,382,034.79 €	− €	− €
GP: follow-up visits	94,182.21 €	233,676.27 €	139,494.06 €	0.10 €
X-ray	7,643,846.81 €	10,884,382.46 €	3,240,535.65 €	2.36 €
Additional diagnostics	27,003,054.78 €	40,026,386.32 €	13,023,331.54 €	9.47 €
Testing costs	22,680,900.00 €	− €	−22,680,900.00 €	−16.50 €
Sum	158,206,727.88 €	214,428,877.40 €	56,222,149.52 €	40.90 €
Cost of illness p.p.	115.09 €	155.99 €		

### Extrapolation of costs

Looking at the national level the costs of illness in Table 4 for ILI patients are 800,339,641 € with clinical judgment and 736,143,912 € with POCT assistance based on the patient numbers of the influenza season 2017/18. Taking age into account, the numbers are 145,108,274 € and 148,066,319 € for children, 440,802,489 € and 429,870,865 for adults, and 214,428,877 € and 158,206,728 € for elderly people (Tables 5–7). In total, there are lower costs of 64,195,730 € when using POCT for diagnosing influenza in ILI patients. The largest cost difference lies with elderly patients, with lower costs of 56,222,149 € with diagnostic support.

## Discussion

Cost-of-illness studies are considered an important evaluation technique in healthcare. They itemize, value, and sum the costs to provide detailed information about the economic burden of an illness. Analyzing and comparing these costs can be beneficial, especially for healthcare decision-makers, by providing important information to support the political process management functions in defining and prioritizing the health policies and interventions that are planned to be implemented. This study demonstrates that POCT in primary care in Germany produces lower costs of illness than relying on clinical judgment alone. The savings amount to 64,195,730 € for a season with 9,107,800 ILI patients or 7.05 € per patient. In the subgroups of patients with a higher risk of hospitalization, such as elderly people, early testing at the point of care has a more significant impact on reducing healthcare spending and resource utilization.

The present study was limited by the fact that most of the relatively few studies looking at the consequences of introducing POCT in primary care neglect diagnostic accuracy and do not distinguish between test results and true infection status as confirmed by real-time PCR in the case of influenza. This limitation becomes especially relevant when discussing the cost effects of POCT in general without considering the testing technology (lateral flow or NAAT), which in return has a high impact on the accuracy of the diagnosis. Furthermore, the majority of the studies included in the calculation were not conducted in Germany. As described in the methods section, studies were checked for applicability to the model. However, the German healthcare system has some peculiarities, especially in the primary care sector. Many specialized doctors, such as cardiologists or pulmonologists, have their own offices independent from a hospital. Patients can go directly to these specialists without seeing their GP first and can thus receive specialist-level care without going to a hospital. This could mean that hospitalization rates in other countries do not necessarily reflect the hospital admission frequency in Germany. On the other hand, there is a financial incentive for German hospitals to admit patients to the wards when they are seen at an emergency department. Other countries have higher discharge rates from emergency care^27,28^. The effects run contrariwise and might cancel each other out. This, however, is uncertain and remains a limitation of the results. A possible expansion of the model would be to also look at other respiratory viruses, such as RSV or SARS-CoV-2. In addition to the medical benefits, there could also be an additional economic upside: since the underlying technology of detecting the viruses is the same as that for detecting influenza, there could be synergies in device usage and hence testing costs.

In conclusion, using rapid molecular POCT to support primary care physicians in detecting influenza in ILI patients reduces the overall cost of illness in Germany. This is most significant when used for elderly patients. Fewer follow-up GP visits, fewer additional diagnostics, and a reduction in hospitalizations contribute to lower costs. Decision-makers should also consider other potential benefits that result from POCT, including a positive effect on antibiotic resistance due to fewer prescriptions as well as positive effects on labor productivity due to a shorter period of symptoms with early antiviral treatment. Correct diagnosis using early POCT may also result in more patients receiving the correct treatment. Therefore, from a societal perspective, the benefits of POCT are probably much higher than the present estimations suggest.

## Data Availability

The datasets generated and/or analyzed during the current study are publicly available, previously published data obtained online. The study did not use any original data but used systematic literature on the database MEDLINE to seek suitable publications. The inclusion criteria are specified in the methods. All publications that the calculation and the study are based on are listed in the references section.
